# Increased presence of mammal-eating killer whales in the Salish Sea with implications for predator-prey dynamics

**DOI:** 10.7717/peerj.6062

**Published:** 2018-12-04

**Authors:** Monika W. Shields, Sara Hysong-Shimazu, Jason C. Shields, Julie Woodruff

**Affiliations:** Orca Behavior Institute, Friday Harbor, WA, United States of America

**Keywords:** Killer whales, Habitat usage, Predator-prey dynamics, Salish Sea, Harbor seals, Predator management, Population growth

## Abstract

The inland waters of Washington State and southern British Columbia, collectively known as the Salish Sea, comprise key habitat for two regional populations of killer whales (*Orcinus orca*): the mammal-eating West Coast Transients and the endangered fish-eating Southern Residents. These two populations are genetically distinct and may avoid each other. Transient killer whale usage of the Salish Sea has been previously assessed over two seven-year time periods, showing an increase from 1987 to 2010. We documented a continued significant increase in mammal-eating killer whale presence in the Salish Sea from 2011 to 2017, with intra- and inter-annual variability and with record sightings in 2017. This continued increase, likely in response to abundant marine mammal prey, is related to both a growing population and an increase in the number of West Coast Transients visiting the area. Additionally, a negative binomial regression shows that absence of Southern Residents is correlated to transient presence. Finally, both populations of killer whales have been linked to regional harbor seal populations; harbor seals are salmonid-eating competitors of the Southern Residents and are prey for the mammal-eating transients. With Southern Residents listed as endangered, culling harbor seals has been proposed as a measure to help in their recovery. With this in mind, we developed an energetic model to assess the minimum number of harbor seals consumed by transient killer whales. Using the actual number of whales present in each age-sex class for each day of the year, we estimate that, at a minimum, transients in the Salish Sea consumed 1090 seals in 2017. This is more than 2% of the 2014 estimated harbor seal population the Salish Sea. The population controlling effects of transient killer whale predation on harbor seals should be considered when evaluating any pinniped management actions in the Salish Sea.

## Introduction

The inland waters of Washington State and southern British Columbia, an area known as the Salish Sea, are home to two ecotypes of killer whale (*Orcinus orca*). The Southern Residents are fish-eating whales feeding predominantly on salmonids; West Coast Transients (also known as Bigg’s killer whales), are marine mammal eaters, preying upon species such as seals, sea lions, and porpoises (Ford et al., 1998). Despite having overlapping geographic ranges, these two ecotypes are not known to interbreed (Morin et al., 2010). While rare aggressive interactions have been documented between the two populations (e.g.,  Ford & Ellis, 1999), usually there are no direct interactions at all. Anecdotal observations by the authors and many others have revealed that when in the same waterway, the mammal-eating killer whales often change course to avoid a direct interaction with the fish-eating killer whales.

Both populations have an overall range that extends from southeastern Alaska to California. For the Southern Residents, the Salish Sea has long been considered their core habitat in the spring through fall with the entire population returning to the area during these months (Osborne, 1999; Hauser et al., 2007). By contrast, the mammal eating killer whales roam much more widely throughout their range and throughout the year (Ford et al., 2013), with most individuals only occasionally seen in the Salish Sea. In recent years, however, both of these patterns have been changing. The Southern Residents have been utilizing the Salish Sea less, particularly in the spring months (Shields, Lindell & Woodruff, 2018). Meanwhile, transient killer whales have expanded their usage of the Salish Sea from a small number of whales visiting primarily in August and September in the late 1980s and early 1990s (Baird & Dill, 1995) to visiting in increasing numbers with a second peak in visitation in April and May as of the mid-2000s (Houghton et al., 2015).

The Southern Resident killer whales were listed as endangered under Canada’s Species At Risk Act in 2003 and the United States Endangered Species Act in 2005 with the three primary risk factors being identified: lack of prey, toxins, and vessel effects. Despite more than ten years of recovery efforts including vessel regulations, designation of critical habitat, and extensive research, their population has continued to decline from 88 whales at the time of listing to 74 whales as of October 2018, as per the Center for Whale Research (http://www.whaleresearch.com). While it is recognized that these three risk factors compound each other, lack of prey is seen as the main road block to their recovery (Lacy et al., 2017). Salmon make up more than 90% of their diet (Ford & Ellis, 2006) with a particular preference for Chinook salmon (*Oncorhynchus tshawytscha*) (Hanson et al., 2010). Meanwhile, while the West Coast Transient population is listed as threatened in Canada, their population has doubled between 1990 and 2012 (Towers, Ellis & Ford, 2012). Usage of the Salish Sea by mammal-eating killer whales has been documented during two previous seven-year time periods from 1987 to 1993 (Baird & Dill, 1995) and 2004–2010 (Houghton et al., 2015), with the increased occurrence in the Salish Sea between these two time periods credited to both an increase in prey and an overall increase in population size.

Within the Salish Sea, the increase in transients and decrease in residents may both be traced at least in part to the recovery of harbor seals (*Phoca vitulina*). Harbor seals in the region were periodically culled as a population control measure primarily to benefit fishermen, and were also commercially harvested for pelts from the 1870s through the 1960s (Zier & Gaydos, 2014; DFO, 2010). After receiving protection in British Columbia in 1970 and the United States in 1972, harbor seals experienced a population increase for the next three decades. In the San Juan Islands, annual harbor seal haul-out counts increased from 852 in 1978 to 3,588 in 1999 (Jeffries et al., 2003). In the entire province of British Columbia, population abundance estimates have grown from 10,000 seals in 1970 to 105,000 seals in 2010 (DFO, 2010). Harbor seal populations began to stabilize in the Strait of Georgia between the mid-1990s and 2010 (DFO, 2010). The last published numbers through 1999 indicated that harbor seal numbers in the US portion of the Salish Sea had also reached carrying capacity (Jeffries et al., 2003). Seasonal annual surveys from 2003 to 2018 using a stratified random survey design and line transect or distance survey methods have shown a decline in harbor seal abundance in Washington’s inland waters at a rate of over 5% per year (S Pearson, WDFW, pers. comm., 2018). As of 2014, the estimated harbor seal abundance in the Salish Sea (US and Canada) was 51,000 (Zier & Gaydos, 2014).

Harbor seals are the primary prey of marine mammal eating killer whales. From 1990 to 2011, harbor seals made up 52% of the West Coast Transient diet across their range (Ford et al., 2013). While harbor seals are generalist predators, they do compete with Southern Residents for salmonid prey; seals consume juvenile and adult salmonids while Southern Residents consume only adults. Recent studies have shown that harbor seal consumption of Chinook salmon has increased from 68 to 625 metric tons in Puget Sound from 1970 to 2015 (Chasco et al., 2017), although they also consume significant amounts of salmonid predators such as hake (Zier & Gaydos, 2014; Lance & Jeffries, 2009). Stated another way, in 2015, harbor seals in inland Washington waters were estimated to consume 8.6 million Chinook salmon, compared to an estimated 83,200 Chinook consumed by Southern Residents in the same geographic area (Chasco et al., 2017). The Southern Residents (as well as many Chinook salmon stocks) are listed as endangered in both the United States and Canada, and have continued to experience a population decline over the last 10 years. As a result, many additional recovery measures are being considered, and culling of harbor seals is again being proposed.

Here we document the continued increase of transient killer whales and their usage of the Salish Sea over the seven years from 2011 to 2017 and compare this to two previous seven-year time periods. We correlate the increase of transient killer whales to the decreasing presence of Southern Residents. Additionally, we estimate a minimum number of harbor seals consumed by confirmed transients in the Salish Sea in 2017, numbers that can and should be used to determine impacts on regional harbor seal populations. We predict that transient killer whales will continue to be an efficient method of harbor seal population control, thus benefiting Southern Resident killer whales long term and precluding the need for a culling program as a means of harbor seal population control.

## Materials and Methods

### Documenting resident and transient killer whale presence in the Salish Sea

**Figure 1:**
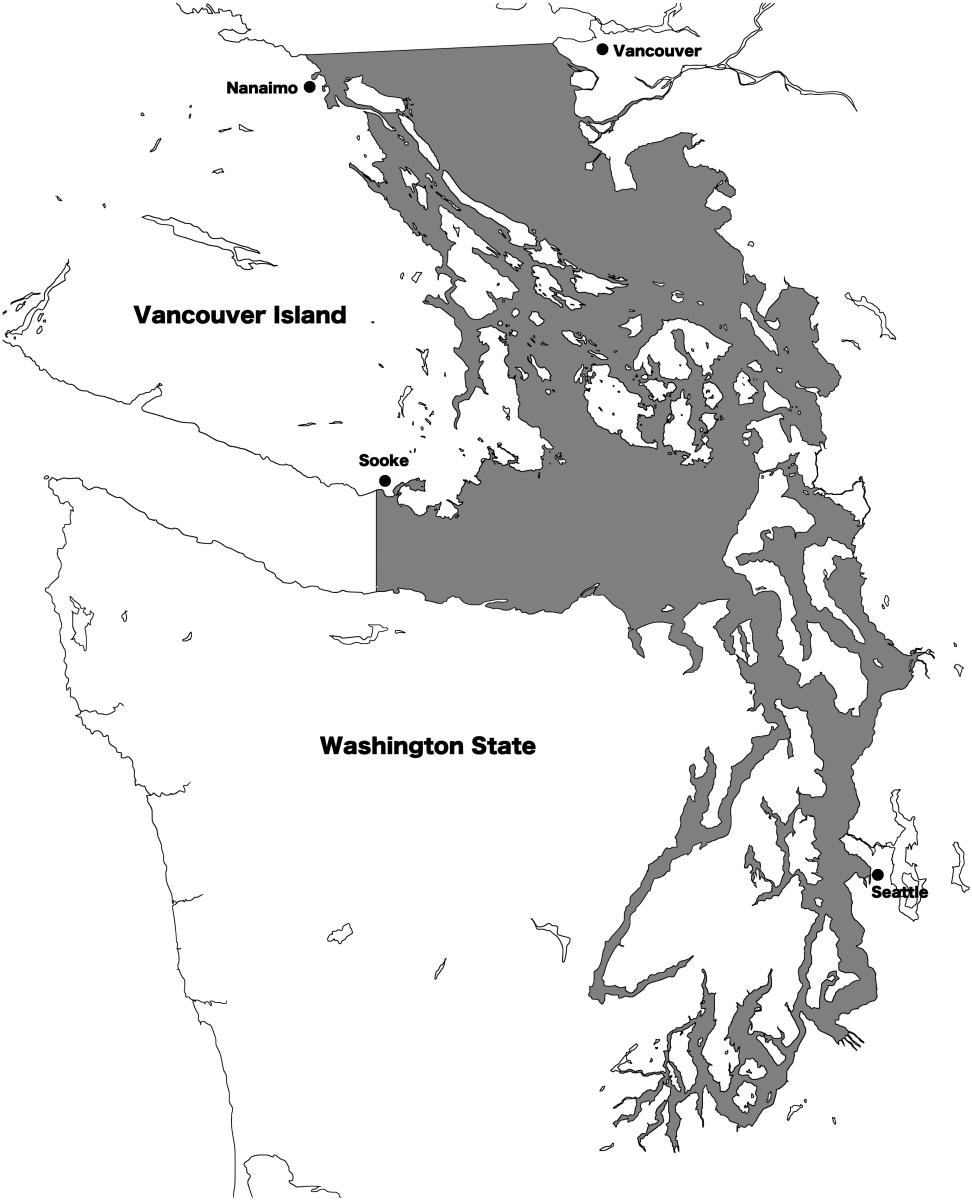
Map of the Salish Sea. The transboundary inland waters of Washington state, USA and British Columbia, Canada are known as the Salish Sea. The shaded region defines the area where killer whale presence was noted for this study.

Resident and transient killer whale presence in the Salish Sea was recorded using 2002–2017 archives from the Orca Network sightings reports (http://www.orcanetwork.org). Since identification skills of the observers providing reports varied widely, we only used identifications by skilled observers. A skilled observer was defined as a researcher, captain, or naturalist from the Pacific Whale Watch Association with more than one year of experience, or a trained shore-based naturalist (from a program such as Whale Scout) with more than one year of experience. Additionally, if photographs of sufficient quality to yield photo-identifications were provided by other observers, these sightings were also included. If identification to “ecotype” (either resident or transient) was not possible, the sighting was discarded. Using number of vessels in the commercial whale watch fleet as a proxy for sighting effort, the fleet size in the region varied year to year from 75 to 95 boats between 2002 and 2015, indicating a slight variation in observer effort over time (Seely et al., 2017). Date, location, and ecotype were recorded, and for transients, confirmed individuals and/or matrilines identified were also recorded based on established photo-ID classifications (Towers, Ellis & Ford, 2012). For the purpose of this study, a transient killer whale matriline was considered to include a female and all of her non-dispersed offspring. All individual killer whales in the region have alphanumeric designations, and matrilines are designated by the name of the adult female that heads the group: for instance, the T124As. The Salish Sea was defined as all the inland waters east of Sooke, British Columbia, and south of Nanaimo, British Columbia, including waters of the Strait of Georgia, Strait of Juan de Fuca, and Puget Sound (Fig. 1).

Following the protocol of Houghton et al. (2015), we further aggregated transient killer whale sightings into “occurrences” for the seven-year time period of 2011–2017. This was done in order to minimize detections of the same group of whales in the area for multiple days, and also to account for whales that may not have been detected on a given day. An “occurrence” was defined as the encounter of a specific social group of transient killer whales when no encounters of that group occurred within the 6 days prior to or after that sighting. For example, if the T65As were seen on three consecutive days, that would count as a single occurrence. If they were gone for two weeks and returned for another two days, that would be a second occurrence. If on the next day they were still present, but were now traveling with the previously unreported T65Bs, that was considered a third occurrence.

Minimum group size was also noted, and was defined as the minimum number of confirmed identifications of whales/matrilines present. In cases of matrilines with consistent membership, the identification of that matriline was considered sufficient to consider all whales of that family group present. In cases where some of the whales are known to disperse from the matriline, the number of whales in the smallest known social grouping of that matriline was used. For example, the T100Bs (three whales as of 2017) sometimes split off from the T100s (four whales as of 2017). If the sighting only reported “the T100s” without specifying whether the T100Bs were present or absent, only 4 whales were added to the minimum group size total.

To test for significant changes in monthly occurrence across the time periods of the three studies, we fit a generalized linear model with a log link function and a negative binomial error distribution to the monthly counts of occurrences with both time period and month as predictors. Pairwise differences between time periods were assessed using a Tukey post-hoc test with *p*-values adjusted for multiple comparisons. The model was fit using the MASS and multcomp packages in R. We used a negative binomial regression model to compare a count of Southern Resident killer whale days in the Salish Sea from April through September between 2002 and 2017 with a count of transient killer whale days over the same time period. In both of the above cases the negative binomial model was used because the data showed overdispersion in the Poisson model.

### Estimating harbor seal consumption

We developed a bioenergetic model of expected harbor seal consumption by individual transient killer whales in the Salish Sea between 1 January 2017 and 31 December 2017. The presence and identity of transient killer whales was recorded using the Pacific Whale Watch Association (PWWA) sightings page, where experienced captains and naturalists report whale locations and identities multiple times per day throughout the year. For the sake of comparison and consistency with historic analyses, this record (often more complete and detailed relative to the public sightings reported to Orca Network) was not used in coding for occurrences, but since it provides a year-round reliable source of whale identities it was well-suited for this purpose. In most cases, whales were identified to the matrilineal level. As above, the minimum number of whales in a social grouping for a particular matriline was used when sub-groups were not specified.

The number of seals (*N*_seals(*i*)_) an individual whale *i* eats per day is a function of its energetic needs (*E*_*i*_), the energetic content of a seal (*K*_seal_), the portion of a transient killer whale’s energy needs that are met by seals rather than other prey (*P*_seal_), and the assimilation rate (the portion of ingested prey absorbed into the body of the predator), which we assume is 0.85 (Williams et al., 2004).

\begin{eqnarray*}{N}_{\mathrm{seals}(i)}= \frac{{E}_{i}\times {P}_{\mathrm{seal}}}{0.85\times {K}_{\mathrm{seal}}} . \end{eqnarray*}

We calculated the energy needs (*E*_*i*_ in kcal/day) of an individual killer whale (*i*) as a function of their mass (M, in kg) based on their age-sex class using a power law (Williams et al., 2004):

\begin{eqnarray*}{E}_{i}=27.91\times \left( 19.65\times {M}_{ \left( i \right) }^{0.756} \right) . \end{eqnarray*}

Individuals were assigned to the age-sex class “immature” if as of 2017 they were under 15 years old, and to either “adult female” or “adult male” for ages 16 and above. Whales over 15 years old but of unknown sex were assumed to be female for the sake of a conservative estimate of bioenergetic needs, as males are significantly larger than females.

Caloric content of seals and the portion of killer whale energetic needs met by harbor seals (Table 1) were calculated from published values of caloric value, mass, and dietary preferences of killer whales for their four primary prey species in the Salish Sea (Baird & Dill, 1995; Ford, 2017; Ford et al., 2013; Hoelzel, 2002; NOAA, 2018; Perez, 1990; Stewart & Clapham, 2002; WDFW, 2012). *K*_*j*_ was calculated as the caloric content per kilogram of prey species *j* multiplied by the average mass of species *j*. From the literature (see Table 1), we obtained estimated prey preferences of West Coast Transient killer whales, expressed as the portion of prey items that are of a given species (*Q*_*j*_). Then, *P*_seal_ was calculated as:

\begin{eqnarray*}{P}_{\mathrm{seal}}= \frac{{K}_{\mathrm{seal}}\times {Q}_{\mathrm{seal}}}{\sum _{j}{K}_{j}\times {Q}_{j}} . \end{eqnarray*}

The number of days a whale was present in the study area times the number of seals it requires per day was then the estimated total number of seals consumed in the study area during the year, and the sum of these numbers across all whales was the total number of seals consumed annually.

**Table 1:** Parameters of killer whale prey species. The top four West Coast Transient prey species with values used in the energetic model, including a range for average mass in kilograms, energetic content in kcal/kg, and estimated proportion of killer whale diet.

Prey species	Average mass in kg^a^	Energetic content in kcal/kg^b^	Portion of West Coast Transient prey items^c^
Harbor seal (*Phoca vitulina*)	65–150	3,550	0.69
Harbor porpoise (*Phocoena phocoena*)	55–70	4,730	0.13
Dall’s porpoise (*Phocoenoides dalli )*	170–200	4,730	0.06
Steller sea lion (*Eumetopias jubatus*)	420–700	2,500	0.12

**Notes.**

a Range, based on Ford (2017), Hoelzel, 2002, Stewart & Clapham (2002) Stewart & Clapham (2002), and WDFW (2012).

b Perez (1990).

c Based on combined data from Baird & Dill (1995) and Ford et al. (2013).

While our search of the literature suggested reasonable ranges for the masses of prey items and killer whales, there is still uncertainty in these values. To account for these values, we performed 10,000 Monte Carlo simulations of our bioenergetic model. In each iteration, values of average prey masses were chosen from continuous uniform distributions with a range based on values found in the literature (Table 1). Individual killer whales were similarly assigned a mass from age-sex class specific ranges based on previously published estimates (Table 2; Baird & Dill, 1995; Ford, 2017; Williams et al., 2004). We then estimated the total seals eaten in 2017 based on the number of days each whale was in the study area, their assigned mass, and the assigned masses of prey items. We then derived 95% confidence intervals, as well as mean estimates, of the number of seals eaten.

**Table 2:** West Coast Transient killer whale mass by age-sex class. Definitions of age-sex classes used in the energetic model with range of masses used in kilograms.

Age-sex class	Definition	Mass (range)^a^
Immature	All individuals <15 years old	1,500–2,000 kg
Adult female	Females or unknown sex ≥ 15 years old	3,000–4,000 kg
Adult male	Males ≥ 15 years old	4,000–8,000 kg

**Notes.**

a Ranges are based on values reported by Baird & Dill (1995), Ford (2017), and Williams et al. (2004).

## Results

**Figure 2:**
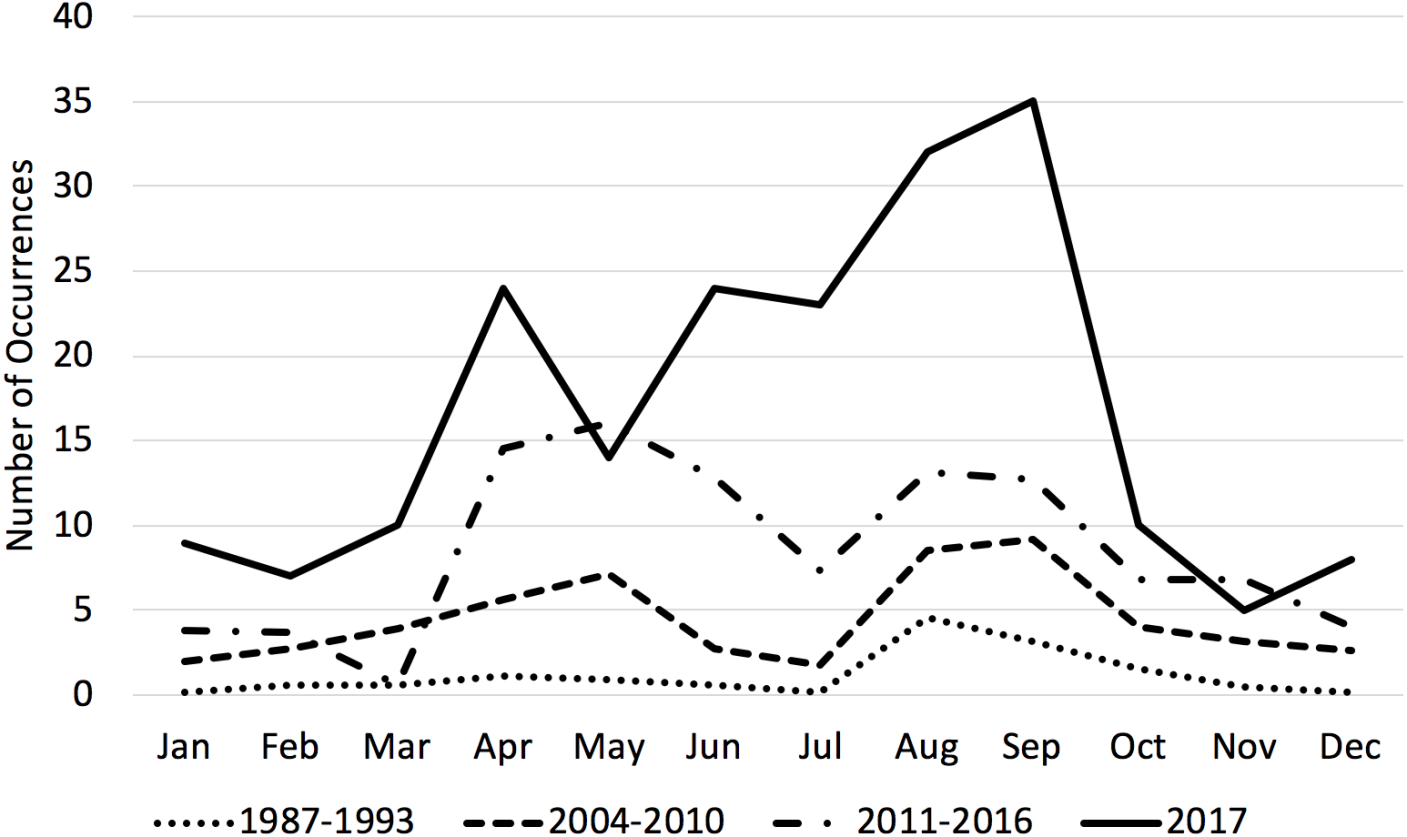
Average number of transient killer whale occurrences by month across three time periods. Historically, transient killer whale occurrences in the Salish Sea peaked in August. By the 2000s, usage of the Salish Sea included a second peak in April–May. By the present time period of 2001–2017, transients were more abundant throughout the entire year, particularly for all the months from April–September. 2017 was a record year for occurrences. 1987–1993 data from Baird & Dill, 1995; 2004–2010 data from Houghton et al. (2015).

Transient killer whale occurrences increased both relative to previous time periods and yearly during the 2011–2017 time period (Table 3). Unlike previous time periods, which had one or two discrete peaks, transient occurrences were steadily high from April through September (with a slight lull in July, see Fig. 2) and higher overall throughout the year compared with historical time periods. Monthly counts of occurrences varied significantly between months and time-periods. A post-hoc test showed that all three time periods were significantly different from one another (*p* < 0.05).

**Table 3:** Transient killer whale “occurrences” across three seven-year time periods. A comparison of occurrences of transient killer whales from 2011–2017 with two previously published seven-year time periods. In addition to the cumulative number of occurrences, number of matrilines, approximate number of total individuals, successful births among whales using the Salish Sea (defined as whales born during the time period that were still alive at the end of the time period), and modal and mean group sizes are also presented.

	# of Occurrences	# of Matrilines	Approximate # of individuals	Successful births among Salish Sea matrilines	Modal group size	Mean minimum group size (±SD, range)
1987–1993 (Baird & Dill, 1995)	97	12	40	4	3	4.40 (2.82, 1–15)
2004–2010 (Houghton et al., 2015)	372	28	135	31	4	5.12 (4.36, 1–36)
2011–2017	854	52 (+18 lone whales)	240	51	4	6.10 (4.45, 1–32)

The most commonly observed group size in the 2011–2017 time period was 4, the same as in the 2004–2010 time period (Houghton et al., 2015). The mean group size in the 2011–2017 time period (mean ± SD: 6.10 ± 4.45, range 1–32) was larger than the mean group size in the 2004–2010 time period (mean ± SD: 5.12 ± 4.36, range 1–36, per Houghton et al., 2015).

Of the 28 matrilines that used the Salish Sea in 2004–2010, by the end of 2017, three of them had gone extinct, six of them were down to a single individual that was still present, and 19 of them continued to be present as matrilines. The increase in number of matrilines using the Salish Sea can be explained by both existing matrilines growing and splitting off (14 new matrilines due to dispersal) and new whales utilizing the Salish Sea (19 known West Coast Transient matrilines newly utilizing the Salish Sea). In addition to the 52 matrilines using the Salish Sea during 2011–2017 there were 18 lone whales, made up of both males and females that either dispersed or were the last of their matriline. This group consisted of both whales that were present in the previous time period and individual whales that were new to the area. Lone whales were not explicitly reported in the earlier time periods.

In addition to an increase in the number of West Coast Transient matrilines using the Salish Sea, the population has also been growing. Among matrilines documented in the Salish Sea during the seven-year time period from 2011 to 2017, 51 successful births occurred, where a successful birth was defined as a whale born during this time period that was still alive at the end of 2017.

**Figure 3:**
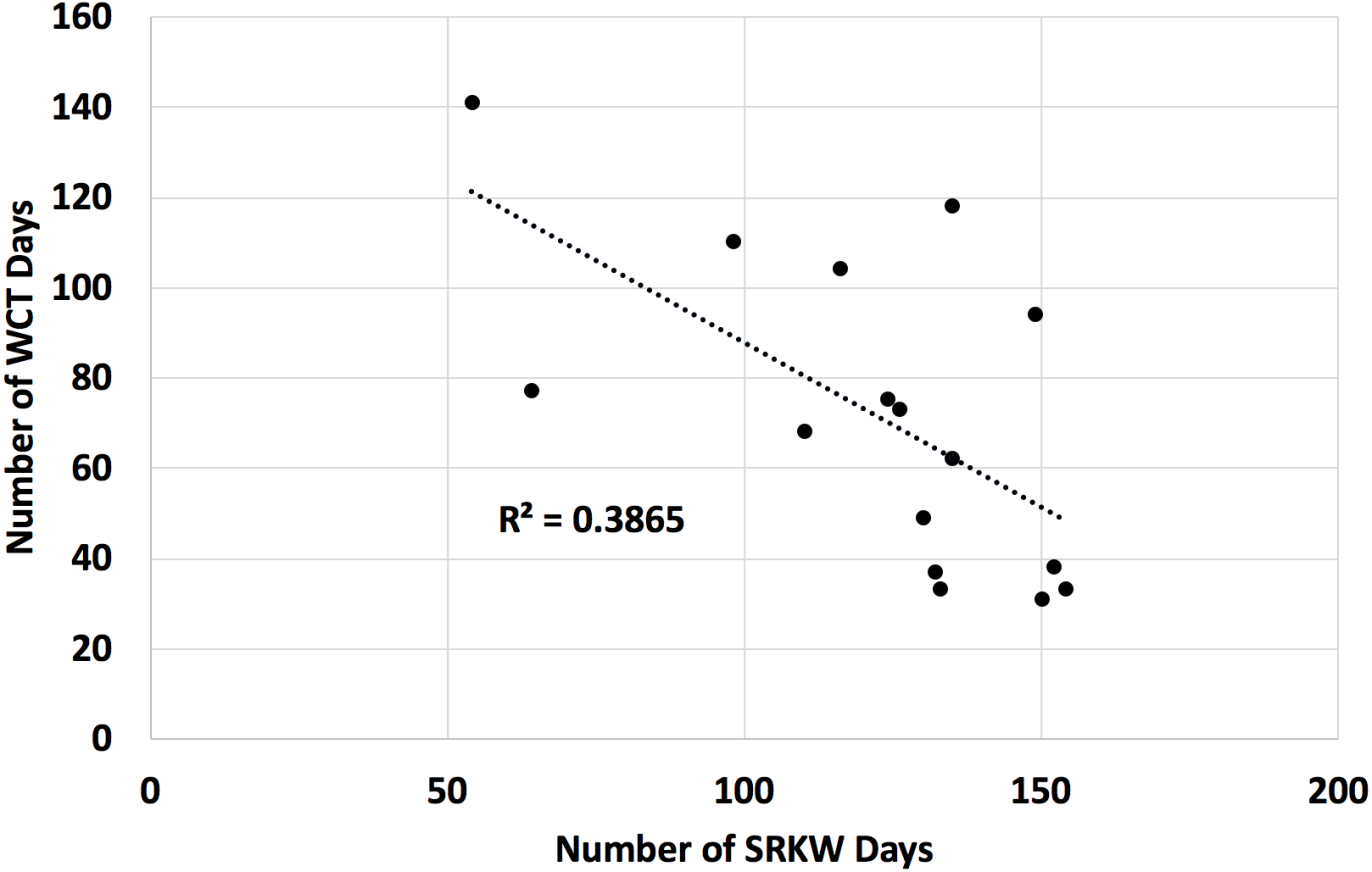
Number of days Southern Resident (SRKW) and transient (WCT) killer whales were present in the Salish Sea in the months of April through September for 2002 through 2017. The Salish Sea has been identified as the core critical habitat for the endangered Southern Resident killer whales in both the United States and Canada, but their usage of the Salish Sea during the months of April–September has declined over the last 16 years. Concurrently, transient killer whale usage of the Salish Sea has increased. Data on presence/absence of both species is from public reports from knowledgeable observers to the Orca Network sightings database. While both these trends are linked at least in part to prey abundance of the respective killer whale populations, the two populations are also known to avoid each other, and transient killer whale presence is correlated to Southern Resident killer whale absence.

We found that a count of Southern Resident killer whale days in the Salish Sea from April through September was a significant inverse predictor of the count of transient killer whale days in the Salish Sea over the same time period (Fig. 3, negative binomial regression *β* =  − 0.010, *χ*2 = 25.00, *p* < 0.01).

**Figure 4:**
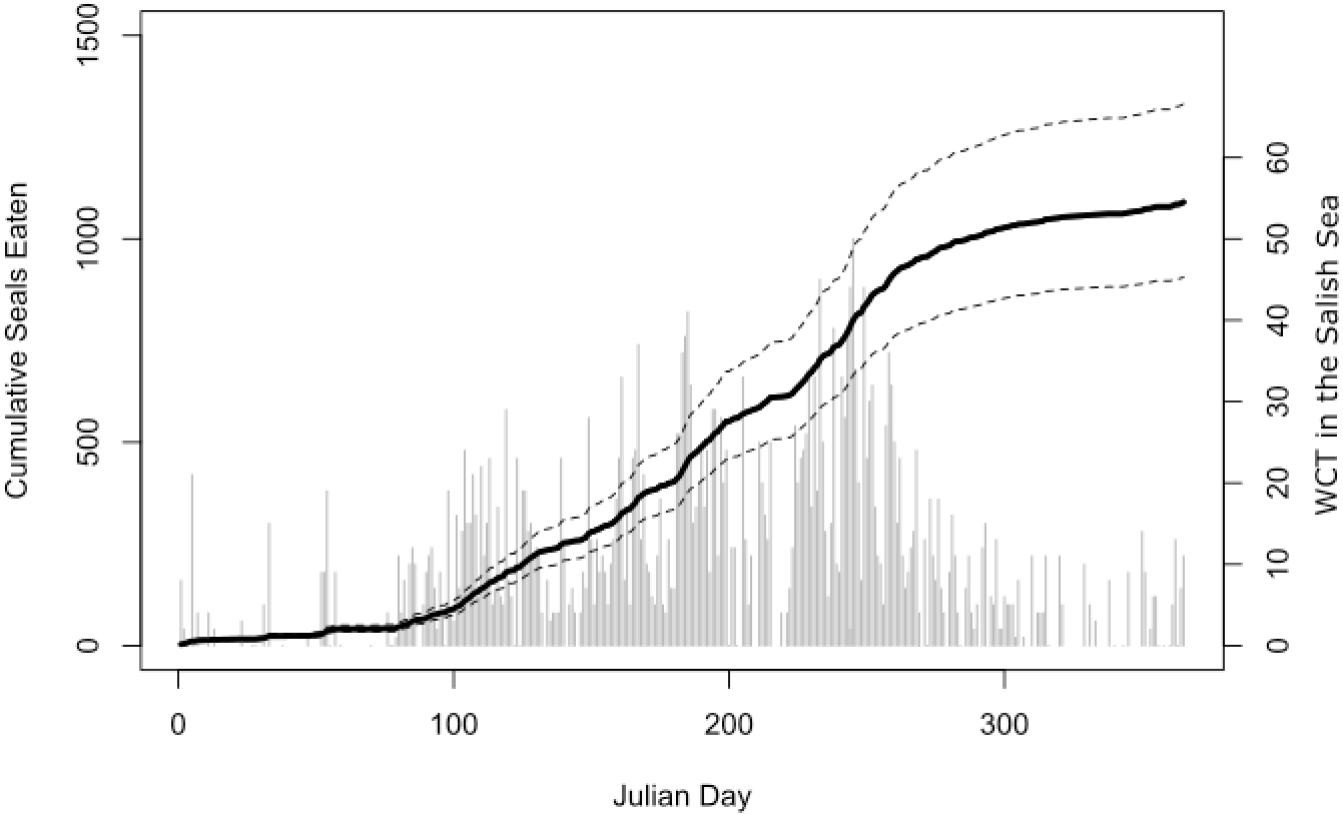
Estimated harbor seal consumption by West Coast Transient (WCT) killer whales in the Salish Sea in 2017. Black line is the mean estimate of cumulative seals eaten (left axis) through that day of the year from our Monte Carlo bioenergetic model, based on actual transient killer whale presence as reported by the Pacific Whale Watch Association. Dashed lines represent 95% confidence intervals. Gray bars are the number of WCT killer whales confirmed in inland waters on each day (right axis).

Our bioenergetic model of harbor seal consumption arrived at a mean estimate of 1,090 seals consumed by killer whales in the Salish Sea in 2017. While there was some uncertainty, our lower 95% confidence interval was still over 900, while our upper confidence interval suggested that over 1,300 seals may have been consumed (Fig. 4).

## Discussion

We documented a continued increase in the number and occurrence of mammal-eating killer whales in the Salish Sea compared to two previous seven-year time periods. During the 1987–1993 time period, there was a noted peak of occurrences in August and September coinciding with harbor seal pupping season. While harbor seal pups are smaller than adults, they are also likely easier to hunt. During the 2004–2010 time period, there was a second peak in April and May. While our data for 2011–2017 show an increase in transient killer whale occurrence in every month of the year, there are still obvious peaks in April–May and August–September, with a slight lull in June–July (Fig. 2). The time of harbor seal pupping varies by latitude, with mid-June being the peak for pupping season in southeastern Alaska and early August being the peak in the Salish Sea (Temte, Bigg & Wiig, 1991). The decline in transient occurrences in the Salish Sea in June and July coincides with a peak in sightings of West Coast Transients in southeastern Alaska, as at least part of the population likely takes advantage of the peak pupping season in both regions (Ford et al., 2013). 2017 was a record sightings year for transient killer whales in the Salish Sea.

Despite harbor seal numbers leveling off in the Salish Sea over the last 15 years (S Pearson, WDFW, pers. comm., 2018), our results show transient killer whale visits to the Salish Sea have continued to increase. We propose three compatible explanations for this. First of all, part of this increase can be attributed to an increasing transient killer whale population. Towers et al. (2018) reported that the population as a whole has doubled in size since 1990. We documented 14 new matrilines using the area due to dispersal, meaning that as their family groups grew larger, they split into smaller groups (likely to increase foraging efficiency, as suggested by Baird & Dill, 1996) resulting in more possible groups to be observed.

Secondly, some of this increase is also due to known West Coast Transient killer whales “new” to visiting the Salish Sea, as evidenced by 19 matrilines present in our study that were not reported by either Houghton et al. (2015) or Baird & Dill (1995). A similar migration of matrilines “new” to using the inland waters of British Columbia was documented in the mid-1970s to mid-1990s, another time period when the West Coast Transient population was experiencing significant population growth and increasing availability of harbor seals (DFO, 2009). Long-term monitoring studies have shown that while most members of the population have been encountered over the wide geographic range of the population as a whole, at least some whales show strong site fidelity to certain areas of the coast (Ford et al., 2013). Referencing data from the most commonly encountered transient killer whales (>50 encounters between 1990 and 2011) from Ford et al. (2013), it appears that whales that previously frequented higher latitudes (i.e., southeastern Alaska) make up many of the whales that are now more regularly using the Salish Sea. For example, T86A, a whale not documented in any occurrences in 1987–1993 or 2004–2010, had a mean encounter latitude of over 58° from 1990 to 2011. Between 2011 and 2017, she was part of 28 occurrences in the Salish Sea, which is centered around a latitude of 48° , indicating she has expanded her regular range by at least 1100 km to the south. More whales from the population may be using the Salish Sea in delayed response to the increased abundance of harbor seals; a time lag in predator response has been observed in other well-studied predator- prey dynamics (e.g., lemming predators in the Arctic, Gilg et al., 2006). There is also some evidence that different groups of transient killer whales specialize on different types of prey and/or different foraging techniques (e.g., Baird & Dill, 1995), so new matrilines may be moving into the Salish Sea to take advantage of other marine mammals. Harbor porpoise, for instance, have been steadily increasing in Washington inland waters over the last 15 years (Jefferson et al., 2016; S Pearson, WDFW, pers. comm., 2018) This could help explain the unprecedented in sightings in 2017. This boom year does not appear to be an anomaly, as sightings were even higher through the summer of 2018.

Thirdly, a possible explanation is that the transients are continuing to move in to the Salish Sea in part because the Southern Residents are utilizing this habitat less. While there is no competition for prey resources between these two populations, anecdotal observations of mammal-eating transients avoiding fish-eating residents have been regularly made over the 50 years of killer whale study in the region. There is still much more to be understood about why and how the Southern Residents and West Coast Transients avoid each other while sharing the same habitat, but our data show that the abundance of prey coupled with the absence of the Southern Residents may have made the Salish Sea an increasingly attractive habitat to the growing West Coast Transient population.

With more transient killer whales spending more time in the Salish Sea, they are consuming a significant proportion of the regional marine mammal populations, particularly their primary prey: the harbor seal. With a 2014 estimate of 51,000 harbor seals in the Strait of Georgia and Washington’s inland waters (Zier & Gaydos, 2014), transients consumed (at a minimum) more than 2% of the regional harbor seal population in 2017 alone. By taking a minimum of 1,090 seals in 2017, transient killer whales are exceeding the approximately 1,000 seals per year killed in Washington State between 1943 and 1960 (Newby, 1973). Since we were conservative in all of our parameter estimates, it is likely that the actual number of seals taken far exceeds 1,090, perhaps substantially. In 2017, the authors and others observed groups of transient killer whales taking multiple harbor seals at multiple seal haul outs over the course of less than two hours.

Culling harbor seals has been recently proposed as a recovery measure to benefit Southern Residents since they both prey extensively on Chinook salmon, for instance as a potential management recommendation from Washington State Governor Inslee’s Orca Task Force in 2018 and also by the Pacific Balance Pinniped Society in British Columbia. While culling predators like seals has shown to have an impact on decreasing predator density, studies have been inconclusive as to impacts on prey density (Bowen & Lidgard, 2012). It is easy to point to the increase in regional harbor seal abundance compared to historic lows (Jeffries et al., 2003) as a correlate to declining regional Chinook salmon populations (Pacific Salmon Commission, 2017), but predator-prey dynamics are complex, particularly in cases like these where harbor seals are generalist predators and Chinook salmon are prey to a wide variety of species. In cases such as these, culling can also have unintended ecosystem consequences (Yodzis, 2001). Prior to the culling and hunting in the 20th century, harbor seals long existed in large numbers in the region alongside healthy and abundant salmon populations. There is no obvious reason they can’t continue to do so today, if the other factors that have contributed to salmon population declines are addressed: habitat degradation, loss of access to spawning grounds, unsustainable fishing practices, water contamination, etc. Additionally, the increased presence of transient killer whales in response to the increasing numbers of harbor seals is another predictable and natural population-level response of a predator to its prey.

## Conclusion

Marine mammal-eating transient killer whales have been increasingly using the Salish Sea over the last 30 years. This increase has occurred due to existing matrilines being present more often, new matrilines from the greater population utilizing the region, and growth of West Coast Transient population as a whole. Over the last seven years, transients were present every month of the year in greater numbers than they were historically. This continued increase is likely in response to recovering marine mammal populations in the region, but also may have been influenced by the decline in usage of the Salish Sea by the endangered fish-eating Southern Resident killer whale population, as some observations have suggested that the two ecotypes avoid one another. Both these killer whale populations are further linked through pinniped abundance, particularly harbor seals. Harbor seals are salmonid predators and thus competitors with the Southern Residents, and the top prey item for transient killer whales. As harbor seal numbers reached historic highs in the Salish Sea, transient killer whales have moved in to take advantage of their abundance, consuming at a minimum more than a thousand seals per year given the number of days they were present in 2017. Given the ongoing increase of transients, our conservative estimate of seal predation, and the regional decline of harbor seals, the population-controlling effects of transient killer whales on seals is rapidly increasing. This dynamic should be explored further before interfering with the natural development of this system.

##  Supplemental Information

10.7717/peerj.6062/supp-1 Table S1 SRKW and WCT Presence in the Salish Sea from 2002–2017 Raw data for number of days the Southern Resident and West Coast Transient killer whale populations were in the Salish Sea from the months of April–September between 2002–2017. Data compiled from Orca Network sightings reports.

10.7717/peerj.6062/supp-2 Table S2 Transient Killer Whale Occurrences by Month Across Three 7-Year Time Periods Raw data for total number of occurrences by month across three 7-year time periods. 1987–1993 data from (Baird & Dill, 1995). 2004–2010 data from (Houghton et al., 2015).

10.7717/peerj.6062/supp-3 Supplemental Information 3 Bioenergetic model from transient killer whale seal consumption R code used for the bioenergetic model.

10.7717/peerj.6062/supp-4 Data S1 2017 Transient Killer Whale Presence in the Salish Sea Raw data showing the number of days each transient killer whale was present in the Salish Sea in 2017 per Pacific Whale Watch Association reports. Age-sex class used in the bioenergetic model is also indicated. These values were used to calculate a minimum estimate for number of harbor seals consumed by transients in the Salish Sea in 2017.

## Additional Information and Declarations

### Data Availability

The following information was supplied regarding data availability: Raw data is provided in the Supplemental Files.
